# Application of biofilm dispersion-based nanoparticles in cutting off reinfection

**DOI:** 10.1007/s00253-024-13120-7

**Published:** 2024-06-19

**Authors:** Xiaojuan Li, Shiyu Lin, Yueli Wang, Yang Chen, Wei Zhang, Gang Shu, Haohuan Li, Funeng Xu, Juchun Lin, Guangneng Peng, Hualin Fu

**Affiliations:** Innovative Engineering Research Center of Veterinary Pharmaceutics, Department of Pharmacy, College of Veterinary Medicine, Sichuan Agricultural University, Chengdu, 611130 Sichuan China

**Keywords:** Bacterial biofilm, Biofilm dispersants, Dispersed bacteria, Dispersant-based nanoparticles

## Abstract

**Abstract:**

Bacterial biofilms commonly cause chronic and persistent infections in humans. Bacterial biofilms consist of an inner layer of bacteria and an autocrine extracellular polymeric substance (EPS). Biofilm dispersants (abbreviated as dispersants) have proven effective in removing the bacterial physical protection barrier EPS. Dispersants are generally weak or have no bactericidal effect. Bacteria dispersed from within biofilms (abbreviated as dispersed bacteria) may be more invasive, adhesive, and motile than planktonic bacteria, characteristics that increase the probability that dispersed bacteria will recolonize and cause reinfection. The dispersants should be combined with antimicrobials to avoid the risk of severe reinfection. Dispersant-based nanoparticles have the advantage of specific release and intense penetration, providing the prerequisite for further antibacterial agent efficacy and achieving the eradication of biofilms. Dispersant-based nanoparticles delivered antimicrobial agents for the treatment of diseases associated with bacterial biofilm infections are expected to be an effective measure to prevent reinfection caused by dispersed bacteria.

**Key points:**

*• Dispersed bacteria harm and the dispersant’s dispersion mechanisms are discussed.*

*• The advantages of dispersant-based nanoparticles in bacteria biofilms are discussed.*

*• Dispersant-based nanoparticles for cutting off reinfection in vivo are highlighted.*

## Introduction

It is well documented that persistent infections caused by bacteria, including periodontitis, urethritis, and pulmonary cystic fibrosis, are usually associated with biofilm formation (Davies 2003; Green and Jones 2022; Ma et al. 2022; Tang et al. 2022). Biofilms are three-dimensional (3D) structures of bacterial communities encapsulated by autocrine extracellular polymeric substances (EPS), also known as “microbial cities,” with tight physical barriers and extensive transport and communication networks (Fang et al. 2020; Karygianni et al. 2020). EPS mainly comprises extracellular polysaccharides, proteins, and extracellular DNA (eDNA) (Karygianni et al. 2020). Physical barrier EPS-protected bacteria require antimicrobials 10 ~ 1000 times higher than planktonic bacteria (Davies 2003; Ji et al. 2022). In addition, EPS can help bacteria evade the body's immune system (Alhede et al. 2014; Scherr et al. 2014; Ramírez-Larrota and Eckhard 2022). All these reasons contribute to the refractory and complex nature of bacterial biofilm infections.

Currently, biofilm dispersants (abbreviated as dispersants) have proven to be widely used as a standard means of removing the physical protective barrier of bacteria, Such as nitric oxide (NO), D-amino acids (D-AA), enzymes, and surfactants(Fleming and Rumbaugh 2017; Verderosa et al. 2019; Jiang et al. 2020). Dispersion is part of the life cycle of Biofilms, and no studies have yet shown that bacteria are resistant to dispersants (Tian et al. 2021). Dispersants usually have weak or no bactericidal effect. Bacteria that escape the biofilm (abbreviated as dispersed bacteria) can cause recolonization and serious reinfection if not removed promptly (Pettigrew et al. 2014; Fleming and Rumbaugh 2018). For example, in a mouse trauma model, enzyme-catalyzed induction of *Pseudomonas aeruginosa* (*P. aeruginosa*) biofilm dispersion leads to massive bacterial entry into the circulation, causing fatal sepsis (Fleming and Rumbaugh 2018), Undispersed *Staphylococcus epidermidis* (*S. epidermidis*) did not cause serious reinfection (Wang et al. 2011). In addition, bacteria colonizing the interior of the biofilm respond to deteriorating conditions (e.g., high cell density, nutrient depletion, toxic waste accumulation, and signal conditioning), triggering dispersion of the biofilm and leading to reinfection (Bridges and Bassler 2019; Rumbaugh and Sauer 2020; Andersen et al. 2021). Most dispersants are often in combination with antimicrobial agents as a promising means of addressing biofilm reinfection. The insoluble nature of both and their sensitivity to the host immune system make it difficult for them to exert synergistic effects at the site of infection, thus limiting their clinical application (Tian et al. 2021). The desired result is achieved by increasing the corresponding dose, but high doses often lead to side effects (e.g., neurotoxicity, allergic reactions, and liver toxicity) (Bangert and Hasbun 2019). With the continuous development of nano-delivery technology, it has been widely evaluated in improving the solubility of refractory drugs, protecting and hiding Unsteady able medicines, and enhancing drug targeting (Li et al. 2021; Wang et al. 2021; Thorn et al. 2021). So far, there are no detailed reports on the role of dispersants and antimicrobial agents in eradicating bacterial biofilm infections and avoiding reinfection via nano-drug delivery systems (NDDS). This article aims to introduce the causes of harm from dispersed bacteria; the Mechanism of dispersant-mediated biofilm dispersion; the advantages of dispersant-based nanoparticles; clinical applications of dispersion-based nanoparticle-delivered antimicrobial agents in diseases associated with bacterial biofilm infections. It provides an effective means of eradicating bacterial biofilms and avoiding reinfection.

## Causes of reinfection from dispersed bacteria

**Fig. 1:**
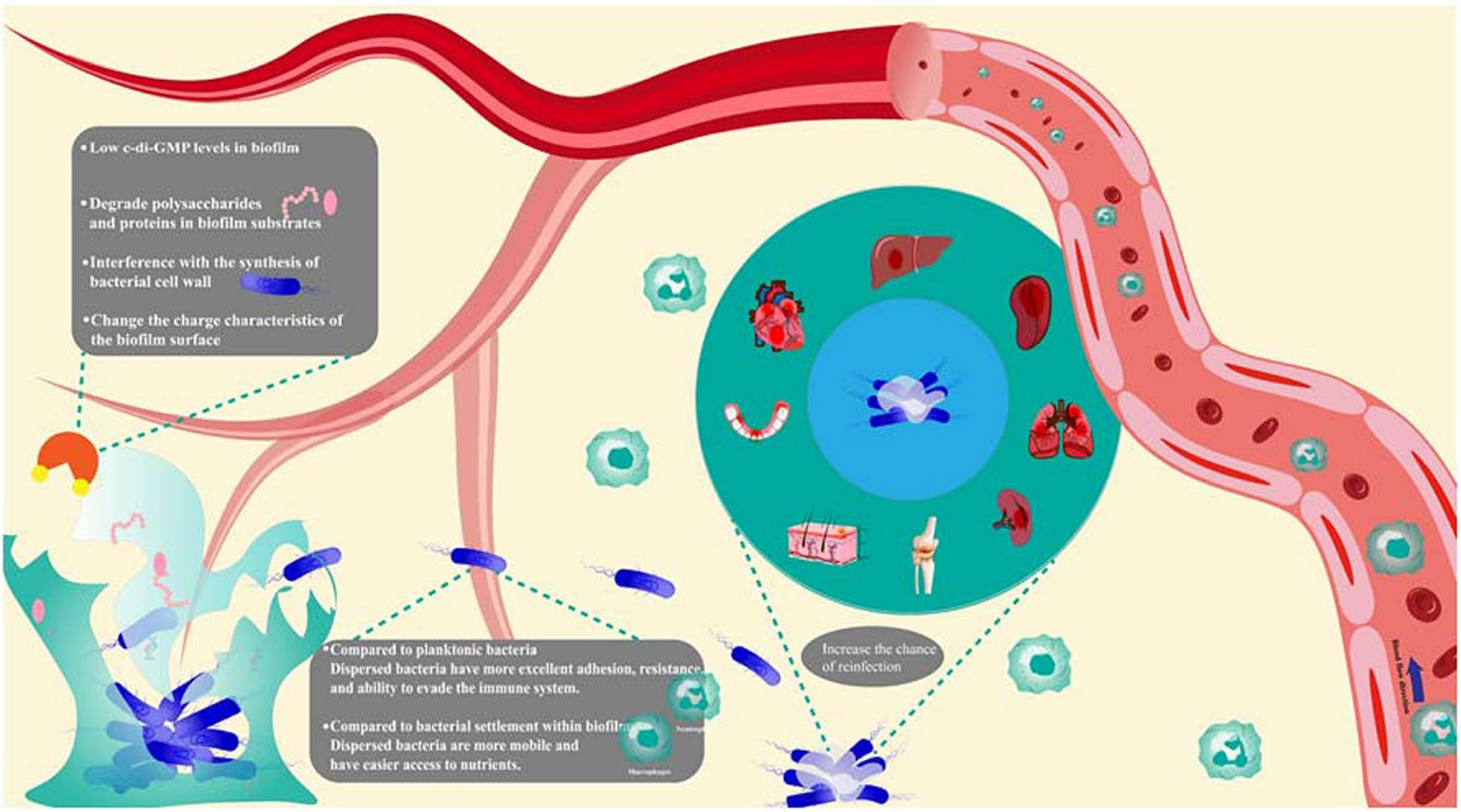
Schematic diagram of the harm caused by dispersed bacteria

Dispersed bacteria are a distinct group of bacteria, different from bacteria settled in biofilms or planktonic bacteria. Compared to planktonic bacteria, dispersed bacteria are more virulent, mainly due to increased adhesion, motility, and resistance. It is also more motile and has easier nutrient access than bacteria that settle inside biofilms. These characteristics of dispersed bacteria can subsequently mean more acute severe infections for the host (Fig. 1).

### Enhanced virulence

Genes encoding virulence-related genes upregulate when dispersed bacteria escape from the biofilm (Fig. 1). Gene transcript expression profiles showed dispersed *P. aeruginosa* virulence-related genes (e.g., secB) upregulate (Chua et al. 2014). In addition, dispersed *Candida albicans* (*C. albicans*) virulence-associated genes (e.g., SAP) upregulate and are not expressed in planktonic bacteria (Uppuluri et al. 2018). SAP has multiple pathogenic functions, such as high adhesion to tissues, invasion, and evasion of the host immune system (Hube and Naglik 2001). The corresponding virulence of dispersed *Klebsiella pneumoniae* (*K. pneumoniae*) is also enhanced and resistant to the phagocytosis of immune cells (Guilhen et al. 2019). Biofilm dispersion is also associated with flagellar formation. For example, *P. aeruginosa* encodes flagellum-associated genes that are upregulated and have enhanced motility compared to biofilm bacteria (Cai et al. 2020). In addition, the study showed that dispersed *Streptococcus mutants* (*S. mutants*) also showed increased virulence, mainly in the form of increased adherence, which was 4 times higher than that of planktonic bacteria (Liu et al. 2013). In summary, dispersed bacteria are generally present with increased invasiveness, motility, and adhesion, increasing the chances of further transmission and reinfection.

### Enhanced resistance

There may be a corresponding increase in the resistance of dispersed bacteria compared to planktonic ones (Fig. 1). Dispersed bacteria are involved in the upregulation of acid-regulated gene (atpD) expression compared to planktonic *S. mutants*, which may enhance acid resistance by participating in intracellular proton pumping (Liu et al. 2013). In addition, dispersed bacteria showed more drug resistance compared to planktonic bacteria. Dispersed bacteria are less sensitive to chlorhexidine (CHX) (Liu et al. 2013). Dispersed bacteria may also have a more robust detoxification capacity than planktonic bacteria. The cusCFBA manipulator in the CH1034 genome overexpressed in dispersed *K. pneumoniae* (Guilhen et al. 2016). The study showed that the cusCFBA manipulator encodes the efflux pumps associated with copper and silver ion detoxification in *Escherichia coli *(*E. coli*) (Chacón et al. 2014). As a result, dispersed bacteria are usually more resistant than planktonic bacteria, making treatment more difficult.

### Enhanced metabolic capacity

Dispersed bacteria have different metabolic capabilities compared to bacteria settled within biofilms and planktonic bacteria (Fig. 1). Genes acquiring zinc, iron, and amino acids in the same nutrient-rich environment significantly upregulate in dispersed *C. albicans* (Uppuluri et al. 2018). In addition, an increase in the activity of the dispersed *S. mutants* phosphotransferase system PTS. It utilizes carbohydrates, such as sucrose, glucose, and mannose, more efficiently than planktonic and bacteria settled within biofilms (Liu et al. 2013). Similar characteristics were demonstrated in influenza A virus (IAV)-induced *K. pneumoniae* dispersion (Pettigrew et al. 2014). These suggest that dispersed bacteria have a high capacity to acquire nutrients and facilitate adaptation to harsh environments.

## Dispersion mechanisms of different types of dispersants

Although biofilms provide a natural defense barrier for bacteria, preventing the penetration of antimicrobial agents and evading the body’s immune defenses, bacteria inhabiting different locations within the biofilm structure can respond to deteriorating conditions, leading to the dispersal of biofilm bacteria (Rumbaugh and Sauer 2020). In addition, the physical barrier to bacteria can be removed clinically by dispersants, such as dispersing signal molecules, EPS removal molecules, and chelating molecules, which promote the dispersion of biofilms (Table 1).

**Table 1:** Dispersion mechanisms of different types of dispersants

Dispersant types	Mediated dispersion mechanism	Applications
NO	Produces cytotoxic nitrogen oxides and reduces c-di-GMP levels (Barraud et al. 2009)	Implant infection Wound infection, Pneumonia (Fei et al. 2020; Liu et al. 2020; Cai et al. 2021)
Fatty acids	Regulation of c-di-GMP levels (Dow et al. 2003; Tao et al. 2010)	− −
Rhamnolipid	May be associated with reduced c-di-GMP levels (Harmsen et al. 2010)	*Helicobacter pylori* gastritis (Shen et al. 2020)
Biological enzymes	Degradation of extracellular polysaccharides, proteins, and eDNA in EPS (Thallinger et al. 2013; Thorn et al. 2021)	Endocarditis, Wound infection, Orthodontic appliances, Pneumonia (Bayer et al. 1992; Fleming et al. 2017; Xie et al. 2020; Delfino et al. 2021)
Nano Enzyme	Generation of ROS to degrade EPS (Vatansever et al. 2013; Ji et al. 2016; Wang et al. 2017)	Wound infections, Implant infections, Subcutaneous abscesses, Pneumonia, Oral infections (Ji et al. 2016; Wang et al. 2017, 2020; Zhu et al. 2021; Hu et al. 2022)
Chelated molecules	Binding c-di-GMP, chelating metal ions (Banin et al. 2006; Ma et al. 2011a; Ammons and Copié 2013)	Pneumonia, Oral infection (Rofeal et al. 2020; Velliyagounder et al. 2018)
D-amino acids (D-AA)	Interference with bacterial cell wall synthesis (De Pedro et al. 2003; Kolodkin-Gal et al. 2010; Pidgeon and Pires 2017)	Pneumonia, Embedded medical device surfaces, Implant infections (Chen et al. 2019; Huang et al. 2020; Fan et al. 2021)
Cationic substances	Electrostatic interactions (Strempel et al. 2014; Tamara et al. 2018; Khan et al. 2020)	Wound infection, Oral infection (Zhu et al. 2021; Lin et al. 2021;)

### Regulation of c-di-GMP levels

#### Nitric oxide (NO)

NO in nature is considered an essential dispersal signaling molecule in biological systems. Because of the short half-life of NO and the difficulty in controlling the release of NO, NO donors such as sodium nitroprusside (SNP), SNO, s-nitrosothiols (rnos), and n-diazobenzenedicarboxylic acid (NONOates) have been investigated (Wang et al. 2002; Dong et al. 2019; Kulbir et al. 2021). According to the study, NO can mediate bactericidal and EPS decomposition through reactive nitrogen oxides (e.g., ONOO-) (Yang et al. 2018). In addition, At lower levels, NO can induce biofilm diffusion by activating phosphodiesterase (PDE) to reduce c-di-GMP levels (Barraud et al. 2009). For example, SNP causes biofilm diffusion in *P. aeruginosa* by decreasing c-di-GMP levels (Barraud et al. 2006, 2009; Cai and Webb 2020). The study showed that NO is also involved in bacterial motility and regulates the dispersal of bacterial biofilms. For example, endogenous NO production by Vibrio cholerae also stimulates the retraction of MSHA hairs to mediate biofilm dispersion (Hughes et al. 2022). NO, as an essential dispersion signal, has been widely used (Table 1).

##### Fatty acids

Initially, fatty acid dispersion signaling molecules (DSF) was identified in *Xanthomonas campestris* as regulating bacterial motility, biofilm formation, and dispersion (Ryan and Dow 2011; Zhou et al. 2017). Cis-2-decanoic acid (cis-DA) from the DSF family is a signaling molecule produced by* P. aeruginosa* that induces the dispersion of various microbial biofilms (Davies and Marques 2009). Cis-DA-induced dispersion was associated with regulating c-di-GMP levels, a finding confirmed in biofilms of wild *Xanthomonas campestris* (Table 1) (Dow et al. 2003; Tao et al. 2010). The cis-DA trans isomer 2 heptylcyclopropane-1-carboxylic acid (2CP) prevents the conversion of 2CP to the active low trans conformation T_2_DA due to the presence of cyclopropanation bonds. The ability to disperse *Staphylococcus aureus* (*S. aureus*) and *P. aeruginosa* biofilms were significantly more robust than cis-DA and T_2_DA (Harrison et al. 2021). DSF is expected to become a common means of dispersing biofilms.

#### Rhamnolipid (RHL)

RHL is a biosurfactant found in the biofilm of *P. aeruginosa* with functions of maintaining water channels, transporting nutrients and metabolic wastes, and dispersing biofilms, etc. (Boles et al. 2005; Irie et al. 2005; Silva et al. 2017). For example, large amounts of RHL can disperse the biofilm of *P. aeruginosa* and form a central cavity structure (Boles et al. 2005). The study showed that RHL-mediated biofilm dispersion of *P. aeruginosa* might be associated with c-di-GMP regulation (Harmsen et al. 2010). In addition, RHL plays a crucial role in regulating the response of *E. coli* biofilms to the dispersion signal N-(3-oxo-dodecanoyl) homoserine lactone (Bhattacharjee et al. 2016). RHL will be an effective reagent for dispersing biofilms (Table 1).

### Effect on target EPS structure

EPS is the main component of biofilms and has various functions, such as preventing drug penetration, evading the immune system, promoting adhesion and aggregation, etc. We are promoting biofilm dispersion by targeting the significant components that make up EPS (e.g., extracellular polysaccharides, proteins, and eDNA) is a commonly used method today.

#### Biological enzymes

Biological enzymes have been shown to disperse biofilms by targeting extracellular polysaccharides, proteins, and eDNA in EPS (Thallinger et al. 2013; Thorn et al. 2021). Extracellular polysaccharides are long-chain polymers linked by glycosidic bonds and play an essential role in maintaining the structure of bacterial biofilms (Fleming and Rumbaugh 2017; Jiang et al. 2020). Glycoside hydrolases (GHs) targeting extracellular polysaccharides in biofilms have become a research hotspot for dispersed bacterial biofilms (Redman et al. 2020). In cystic fibrosis (CF) patients with high alginate content, alginate catabolic enzymes can alleviate lung infections by breaking down alginate (Blanco-Cabra et al. 2020). α-Amylase also achieved similar effects, significantly promoting wound healing (Fleming et al. 2017). Extracellular proteins are the main components that maintain the physical barrier structure (Fleming and Rumbaugh 2017; Jiang et al. 2020). Proteases promote biofilm dispersion by breaking down proteins in EPS and may also disrupt intercellular communication by solubilizing type I signal peptidases. Protease is considered to be an effective method for EPS removal (Schallenberger et al. 2012; Thallinger et al. 2013). For example, serine proteases produced by *S. epidermidis* can eradicate pre-formed biofilms (Martínez-García et al. 2018; Kumari and Sarkar 2018; Weldrick et al. 2019). The eDNA with glue effect integrates bacteria and other components into a 3D framework, which is beneficial in helping to promote bacterial adhesion and gene transfer (Jakubovics et al. 2013; Okshevsky and Meyer 2015; Fleming and Rumbaugh 2017). Deoxyribonuclease (DNase) can specifically target eDNA to promote biofilm dispersion (Thallinger et al. 2013; Jiang et al. 2020). DNase, a marketed mucolytic agent, has been reported to reduce lung infections in CF patients by degrading DNA in sticky sputum (Delfino et al. 2021). The advantage of low off-target probability and high activity of biological enzymes is usually a standard means of causing large-scale dispersion of biofilms. However, biological enzymes are easily deactivated under the influence of pH, ionic strength, or temperature, leading to the rapid development of various nano enzymes.

#### Nano enzymes

In recent years, nanomaterials with enzymatic activity (called nano enzymes) will become environmentally friendly reagents due to their stable structure, low cost, high catalytic activity, and good antibacterial properties (Chen et al. 2018b). Currently, nano enzymes with peroxidase (POD) activity, such as iron oxide, V_2_O_5_, and graphite nitride (g-C_3_N_423_), are reported to catalyze the conversion of H_2_O_2_ to more toxic highly reactive oxygen species (hROS), such as superoxide anion (-O^2−^), hydroxyl radical (-OH) and singlet oxygen (1O2) (Vatansever et al. 2013; Ji et al. 2016; Wang et al. 2017). Inactivates bacteria and breaks down EPS by irreversibly damaging DNA and degrading polysaccharides and proteins (Gao et al. 2014; Ji et al. 2016). In addition, nano enzymes with DNase-like activity, such as cerium (Ce), have been shown to promote DNA hydrolysis (Chen et al. 2016; Hu et al. 2022). Therefore, nano enzymes are expected to be widely used for the catalytic treatment of diseases associated with bacterial biofilm infections (Table 1).

#### Chelating molecules

Chelating molecules trigger biofilm dissipation by binding certain substances within the biofilm, such as c-di-GMP, magnesium, calcium, and iron, etc. (Jiang et al. 2020). BdcA was identified as a regulator of dispersed biofilms, mediating the dispersion of *E. coli*, *P. aeruginosa*, and *Pseudomonas fluorescens* (*P. fluorescens*) by binding to c-di-GMP (Ma et al. 2011a, 2011b). Large amounts of cations, such as magnesium, calcium, and iron, are essential for maintaining the 3D structure of biofilms (Banin et al. 2006; Wang et al. 2019). Lactoferrin (LF) is an antimicrobial agent from the host immune system that has been shown to trigger biofilm dispersion by chelating iron in the iron carrier system and has anti-inflammatory properties (Disease 2009; Alves et al. 2013; Ammons and Copié 2013). LF has been shown to disperse bacterial biofilms, including *P. aeruginosa*, *E. coli*, *Enterococcus faecalis* (*E. faecalis*), and *S. aureus* (Alves et al. 2013; Ammons and Copié 2013). EDTA, 5-nitro-8-hydroxyquinoline, and halogenated phenols (HPs) have also been reported to induce biofilm dispersion by chelating metal ions (Banin et al. 2006; Sobke et al. 2012; Naclerio and Sintim 2021). Therefore, substances required to maintain bacterial biofilm structure through binding are also commonly used to trigger biofilm dispersion.

### Effect on bacterial cell wall synthesis

D-AA is the enantiomer of natural L-amino acids such as D-leucine, D-cysteine, D-tyrosine, etc. D-AA plays an essential role in the formation and dispersion of bacterial biofilms (Kolodkin-Gal et al. 2010; Leiman et al. 2013). D-AA has been shown to have the effect of dispersing biofilms of *S. aureus* and *P. aeruginosa* (Sanchez et al. 2013; Chang et al. 2022). According to the data, exogenous D-AA can integrate into the cell wall by replacing the original peptidoglycan (PG) structural unit. For example, De Pedro et al. found that the original peptidoglycan structural unit is present in the periplasm of *E. coli* and that exogenous D-cysteine integrates the peptidoglycan structure (De Pedro et al. 2003). Pidgeon, S. E. also found similar results (Pidgeon and Pires 2017). The ability of D-AA to inhibit biofilm formation and promote biofilm dispersion is associated with interference with the cross-linking of peptidoglycan chains. D-AAs have significant potential in treating biofilm infections (Table 1).

### Electrostatic interactions

The study showed that cationic substances usually exert their antibacterial and biofilm-damaging abilities through electrostatic interactions (Table 1) (Strempel et al. 2014; Tamara et al. 2018; Khan et al. 2020). Chitosan(CS) is a cationic amino polysaccharide with excellent biocompatibility and biodegradability and is considered an effective antimicrobial and anti-biofilm compound (Khan et al. 2020). The unique antibacterial and antibacterial biofilm properties of CS are often thought to be associated with amines. Positively charged amines cause leakage of cytoplasmic contents and disruption of bacterial biofilm structure through electrostatic counteraction (Tamara et al. 2018; Khan et al. 2020). In addition, most antimicrobial peptides (AMPs) typically exhibit a positive net charge and have similar antibacterial and anti-biofilm mechanisms (Strempel et al. 2014; Yasir et al. 2018). Three bacteriocins, nisin A, lacticin Q, and nukacin ISK-1 can disrupt the membrane potential of Methicillin-resistant *Staphylococcus aureus* (MRSA) within biological membranes, resulting in ATP efflux from bacteria (Okuda et al. 2013). These cationic substances usually exhibit a high degree of permeability. For example, chitosan can disrupt the EPS of many microorganisms to facilitate drug penetration (Orgaz et al. 2011; Ng et al. 2013; Mu et al. 2014). CSA-13 penetrates rapidly into the interior of EPS and penetrates bacteria within *P. aeruginosa* biofilms (Nagant et al. 2013). Therefore, such substances often achieve the ability to penetrate EPS through electrostatic interactions to facilitate the profound delivery of antimicrobial agents to biofilms.

## Advantages of dispersant-based nanoparticles

**Fig. 2:**
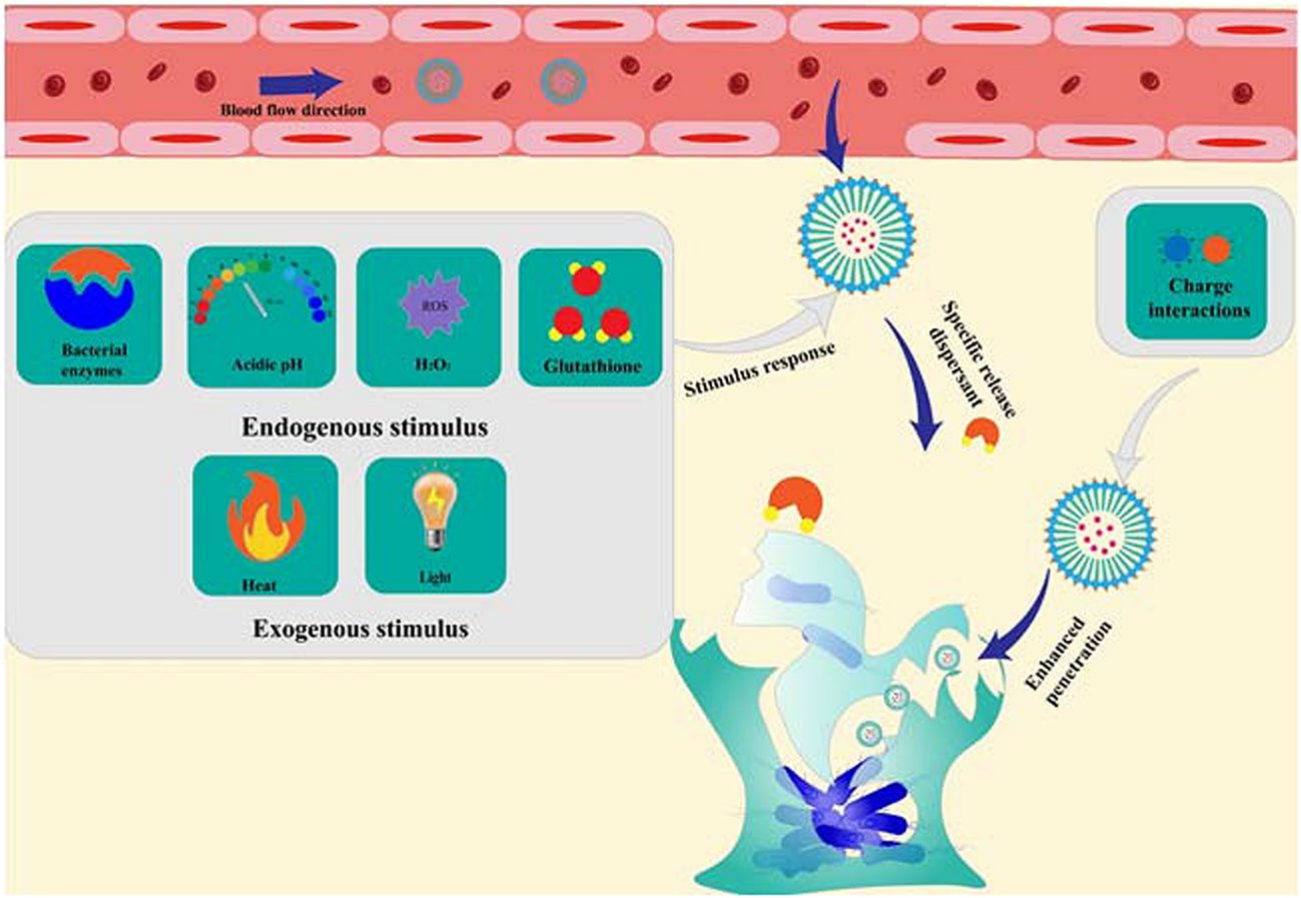
Schematic diagram of the advantages of dispersant-functionalized nanoparticles in dispersing biofilms

Some dispersant-based nanoparticles respond to microenvironmental conditions (e.g., low pH, enzyme overexpression, high glutathione content) and exogenous stimuli (e.g., light) to achieve specific release upon reaching the site of bacterial infection. Dispersants further improve the efficacy of antibacterial drugs by breaking the dense physical barrier and exposing the bacteria inside. In addition, positively charged substances with permeability are often used as carriers or modified on the surface of nanoparticles to deliver antimicrobial agents to biofilms’ interiors effectively. Dispersant-based nanoparticles with specific release and penetration capabilities provide the prerequisites for further improving the antimicrobial effect and avoiding new infections caused by dispersed bacteria (Fig. 2).

### Specific activations

#### Endogenous response

Although the dense EPS provides a rigid physical barrier to bacteria, the bacteria inside the EPS often accumulate large amounts of metabolites in an oxygen-deprived acidic environment (Wang et al. 2013; Fulaz et al. 2019). In addition, the acidic metabolites produced during the inflammatory response increase the acidity of the infected microenvironment. Therefore, acidic microenvironments are often used to design a series of smart responding nanoparticles to trigger the release of dispersants (Fig. 2). pH-sensitive cis-aconitine-D-tyrosine (CA-Tyr) prodrugs bound to polymeric micelle surfaces via electrostatic interactions (Chen et al. 2019; Fan et al. 2021). When this micelle reaches the site of infection, the pH-sensitive cis-aconitine bond breaks, releasing D-Tyr and exposing the positively charged micelle to deliver azithromycin (AZM) efficiently.

A unique high expression of enzymes characterizes the microenvironment of bacterial infection compared to normal tissue (Chen et al. 2018a; Ding et al. 2022). Hyaluronidase (HAS) is a virulence factor highly expressed by Gram-positive bacteria (Hynes and Walton 2000). Hyaluronic acid (HA) is often used as a capping agent to respond to HAS, releasing the protected dispersant (Fig. 2). Qu et al. constructed a smart nanoparticle (AA@GS@HA-MNPs) by coating HA on the sandwich-like graphene-mesoporous silica (GS) loaded with ascorbic acid AA (Ji et al. 2016). HA will be degraded by the HAS secreted by bacteria, releasing H2O2 prodrug AA, catalyzed by MNPs to produce •OH. Compared with GS@HA-MNPs, AA@GS@HA-MNPs significantly dispersed approximately 80% of the biofilm and nearly killed the bacteria embedded in the biofilm. In the presence of HAS, the cumulative release of AA exceeded 73%in 12 h. Successfully established an “on-demand” drug delivery platform. Liu et al. also constructed drug delivery systems that respond to HAS to release dispersants to achieve physical barrier removal and further effective antibacterial (Liu et al. 2019).

Glutathione (GSH) is also present in biofilms, where it is present in concentrations up to 10.0 mM and can protect bacteria from oxidative stress, toxins, and acidity (Gales et al. 2008; Chen et al. 2013; Ding et al. 2022). Nanoparticles that release dispersants in response to this specific microenvironment have been designed (Fig. 2). Alpha-cyclodextrin (α-CD) is conjugated with NO donor through GSH-sensitive bonding to form NO prodrug (α-CD-NO), which then binds to polyethylene glycol (PEG) block peptide copolymer (PEG-(KLAKLAK)2-DA) through host–guest interaction to form smart nanocarriers α-CD-Ce6-NO-DA (Hu et al. 2020). In simulated MRSA biofilms, nanoparticles incubated for 2 h at a concentration of 8 mM GSH released significant amounts of NO but were relatively stable in healthy tissue. The nanoparticles that penetrate the biofilm respond to the overexpression of GSH in the biofilm, leading to a rapid release of NO.

H_2_O_2_ levels at the site of biofilm infection were also higher than in surrounding healthy tissues, mainly due to ROS generated by the host’s immune response (Nathan and Cunningham-Bussel 2013). The study shows that the bacteria's production of H_2_O_2_ comes to interfere with the host's inflammatory response (Erttmann and Gekara 2019). Due to the limitations of H_2_O_2_’s low efficiency, slow onset of action, and high concentration required, it is often catalyzed into hROS to improve bactericidal efficiency. Some nano enzymes with POD activity can catalyze H_2_O_2_ and produce hROS for better antibacterial and anti-biofilm effects (Fig. 2) (Wang et al. 2017; Chen et al. 2018b). Ting Pan et al. designed chitosan-grafted Fe-doped carbon dots nano enzymes CS@Fe/CDs. CD exerts the ability to sterilize and disperse EPS by catalyzing the production of -OH from H_2_O_2_ (Pan et al. 2022). Although the body can release H_2_O_2_ in pathological states, its concentration remains low and does not better stimulate the NDDS to release active substances quickly and efficiently. To increase the level of H_2_O_2_ in the microenvironment, a cascade catalytic reaction generator (CaO_2_/graphene@aluminate) was designed (Yan et al. 2018). First, CaO_2_ can react with water to produce a large amount of H_2_O_2_, which is then catalyzed by graphene to produce hROS, thus destroying biofilm and sterilizing it. According to information, large amounts of H_2_O are potentially toxic to healthy tissue and can delay wound healing (Loo et al. 2012). A specific increase in H_2_O_2_ levels at the site of infection is an effective strategy to reduce toxicity. Currently, a selective increase of H_2_O_2_ levels at the site of infection is often widely used as a reaction condition for the effective release of active substances. Yuting Shi et al. designed enzyme-linked reaction-specific H_2_O_2_-releasing nanoparticles (Shi et al. 2022). In the weakly acidic environment of biofilm infection, the aldehyde condensation bonds of nanoparticles break and release maltose heptose, which is catalyzed by glycosylase (GA) and glucose oxidase (Gox) to produce large amounts of H_2_O_2_, approximately three times more than under physiological conditions. The guanidine group of arginine releases NO under the condition of H_2_O_2_. The microenvironmental response improves the accuracy and release of the dispersant, removing a physical barrier to the further effective performance of the antimicrobial agent.

#### Exogenous response

In recent years, nanoparticles with exogenous conditionally stimulated release dispersants have shown promising results in treating bacterial biofilm infections. Due to their photocontrol ability, high selectivity, and low systemic toxicity have become the ideal exogenous stimulus condition for modulating dispersant release (Fig. 2) (Imberti et al. 2020; Yuan et al. 2020). The dispersant is usually attached to the polymer through a light-crackable joint. Shen, Z. et al. By RAFT polymerization reaction, coumarin chromophore is linked with N-nitrosamines to form (CouNO), which is then coupled with polyethylene oxide (PEO) to form amphiphilic polymer micelles PEO-b-PCouNO spontaneously (Shen et al. 2019). Under visible light, CouNO releases NO spontaneously and releases the loaded Cip when the polymer micelle structure decomposes. The photoresponsive release of NO facilitates removing the physical barrier of *P. aeruginosa*, which improves the prerequisites for the further antibacterial effect of Cip. Duan, Y. et al. also designed similarly functioning light-responsive vesicles (Duan et al. 2021). Visible light-mediated release of NO and gentamicin (GS) has a better anti-biofilm effect than NO or GS alone. In addition, multifunctional nanoparticles with photothermal responsive release dispersants have been developed. Polydopamine (PDA) has received much attention recently as an alternative to photothermal-sensitive materials due to its excellent biodegradability and photo conversion efficiency (Yuan et al. 2020). Yu, S. et al. prepared Fe3O4@PDA@PAMAM@NONOate containing Fe_3_O_4_, PDA, poly(amino) dendrimers (PAMAM), and NONOate (Yu et al. 2018). Under laser irradiation, Fe_3_O_4_ and PDAgenerate heat, increasing the local temperature. NONOate rapidly releases NO, realizing the on-demand triggered NO release under intermittent laser irradiation.

### Strong permeability

EPS, which acts as a physical barrier, severely hinders further penetration of the antimicrobial agent. Positively charged substances can disrupt the intact structure of EPS, are highly permeable, and are often modified on the surface of nanoparticles or used as drug carriers. For example, Ma et al. synthesized CS nanoparticles loaded with curcumin, which enhanced curcumin’s permeability by disrupting biofilms’ structural integrity through electrostatic interactions (Ma et al. 2020). However, the positively charged nanoparticles are easily removed by the body’s immune system and are difficult to reach the infection site through blood circulation. Poly(β-amino) ester (PAE) is a pH-responsive polymer material positively charged by protonation under acidic conditions and is now widely used. PAE tri-block charge reversal micelles for vancomycin delivery prolong the blood circulation time (Chu et al. 2016). Based on in vivo fluorescence imaging results, the 24 h accumulation of charge-reversing micelles at subcutaneous infection sites in mice was 2.7 and 1.8 times greater than vancomycin and positively charged micelles. Charge-reversing nanoparticles that are superior to positively charged nanoparticles are often used to deliver antimicrobial agents. Liu, Y. et al. prepared charge reversal polymer micelles (MSPM) composed of PAE (Liu et al. 2016). Under acidic infection conditions, MSPM positively charged to enhance the permeability of the biofilm, and its permeability was significantly higher than that of triclosan, polymeric micelles composed of triclosan and polyethylene glycol (PEG) (SSPM), resulting in satisfactory therapeutic efficacy. Positively charged nanoparticles with strong permeability effectively deliver antimicrobial agents to the biofilm’s interior, bypassing the biofilm’s resistance to antimicrobial agent penetration.

## Dispersant-based nanoparticle delivered antimicrobial agents in the treatment of biofilm-related diseases

Biofilm is a common cause of various intractable diseases (e.g., traumatic wound infections, lung infections, subcutaneous abscesses, urethritis, etc.) (Mihai et al. 2022). Dispersants can remove the protective shield of bacteria but usually need to use with an antimicrobial agent, which is necessary to eradicate the bacterial biofilm and avoid new infections. The application of dispersants and antimicrobial agents in different forms of NDDS for biofilm-associated infections will be described next.

### Nanoparticles co-modified with dispersants and antimicrobial agents

**Fig. 3:**
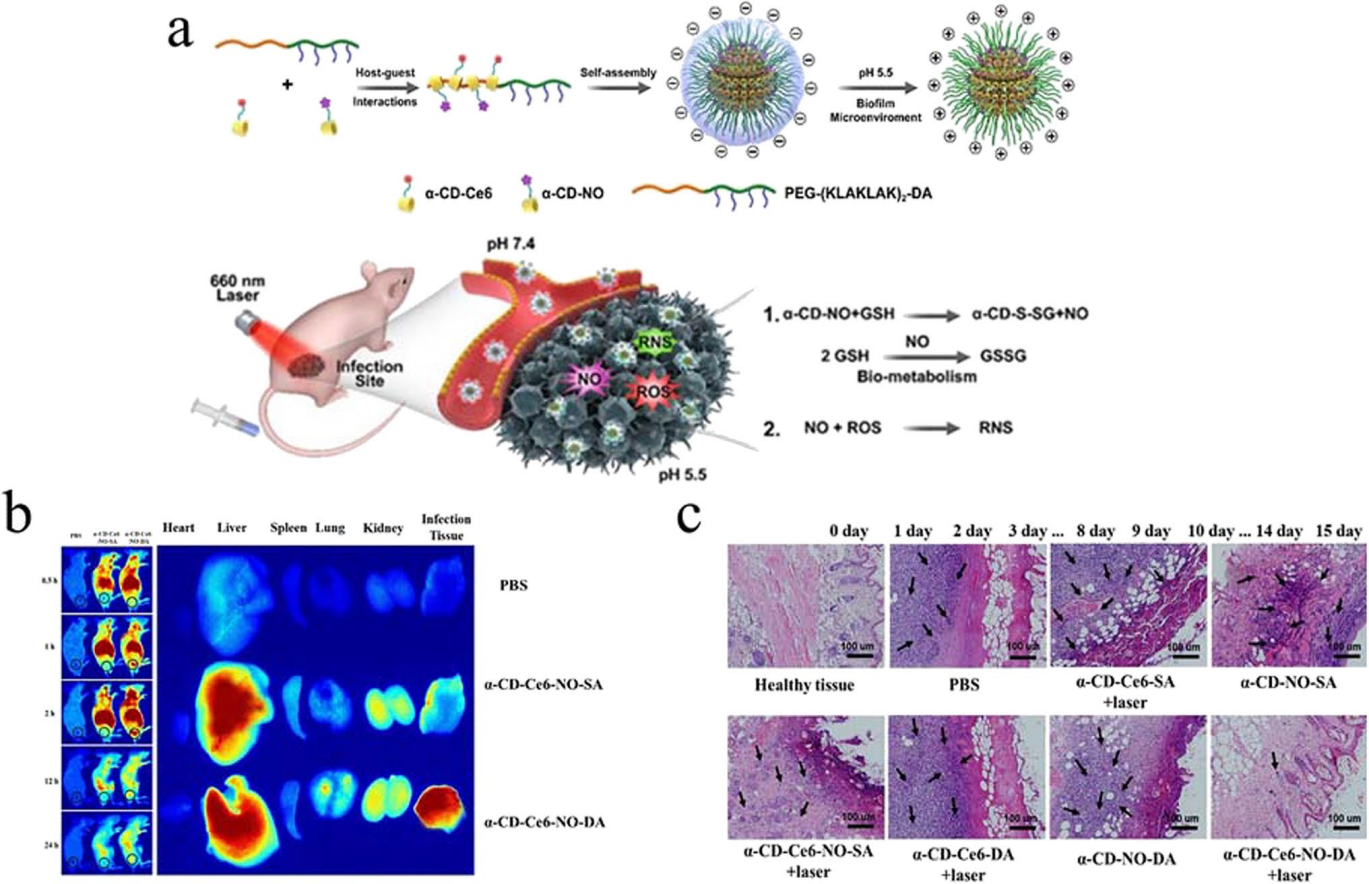
**a** Schematic diagram of the preparation of α-CD-Ce6-NO-DA and the mechanism of MRSA biofilm removal. **b** Fluorescence imaging of MRSA biofilm infection in mice treated with α-CD-Ce6-NO-DA. **c** Histological micrographs of α-CD-Ce6-NO-DA treated infected skin. Reprinted with permission from Ref. (Hu et al. 2020) Copyright 2020, American Chemical Society

NO is an important signaling molecule in dispersing bacterial biofilms. In addition, it also has some ability to promote healing. NO donors co-modified with antimicrobial agents on nanoparticles have achieved the expected results in treating bacterial biofilm-associated infections. For example, azide-modified CS binds to PAMAM via an electroshock reaction, introducing abundant primary amines to load methicillin (Met) and NONOates (Liu et al. 2020). Under physiological conditions, NO is continuously released, along with antimicrobial agents. This delivery system resulted in over 85% dispersion of MRSA biofilm, significantly killing biofilm bacteria. On day 7 of treatment for wound infection, MRSA was barely observable in the wound exudate, demonstrating the fastest rate of wound healing and preventing persistent severe wound infection. Previous researchers have successfully designed specific stimulus-responsive nanoparticles to enhance the release of dispersants and antimicrobial agents. Alpha-cyclodextrin (α-CD) is conjugated with NO donor through GSH-sensitive bonding to form NO prodrug (α-CD-NO), which then binds to polyethylene glycol (PEG) block peptide copolymer through host–guest interaction to create smart nanocarriers α-CD-Ce6-NO-DA (Fig. 3a) (Hu et al. 2020). The nanocarriers are positively charged by charge reversal under acidic conditions, which promotes effective permeation of the nanocarriers within the biofilm. The nanoparticles permeating the biofilm responded to overexpressed GSH, triggering a rapid release of NO, while Ce6 responded to light to produce ROS (Fig. 3a). NO has active nitrogen by reacting with ROS and exerts the desired effect in combination with photodynamic therapy (PDT). In vitro fluorescence imaging showed that the fluorescence of nanoparticles was enhanced 1 h after intravenous injection and stayed at the infected site for 24 h (Fig. 3b). Under the light, it enhances nanoparticles’ antibacterial effect and promotes earlier scarring and faster wound healing (Fig. 3c).

### Dispersant modified antimicrobial nanoparticles/nanoparticles loaded with antimicrobial agents

#### Enzyme modified nanoparticles

**Fig. 4:**
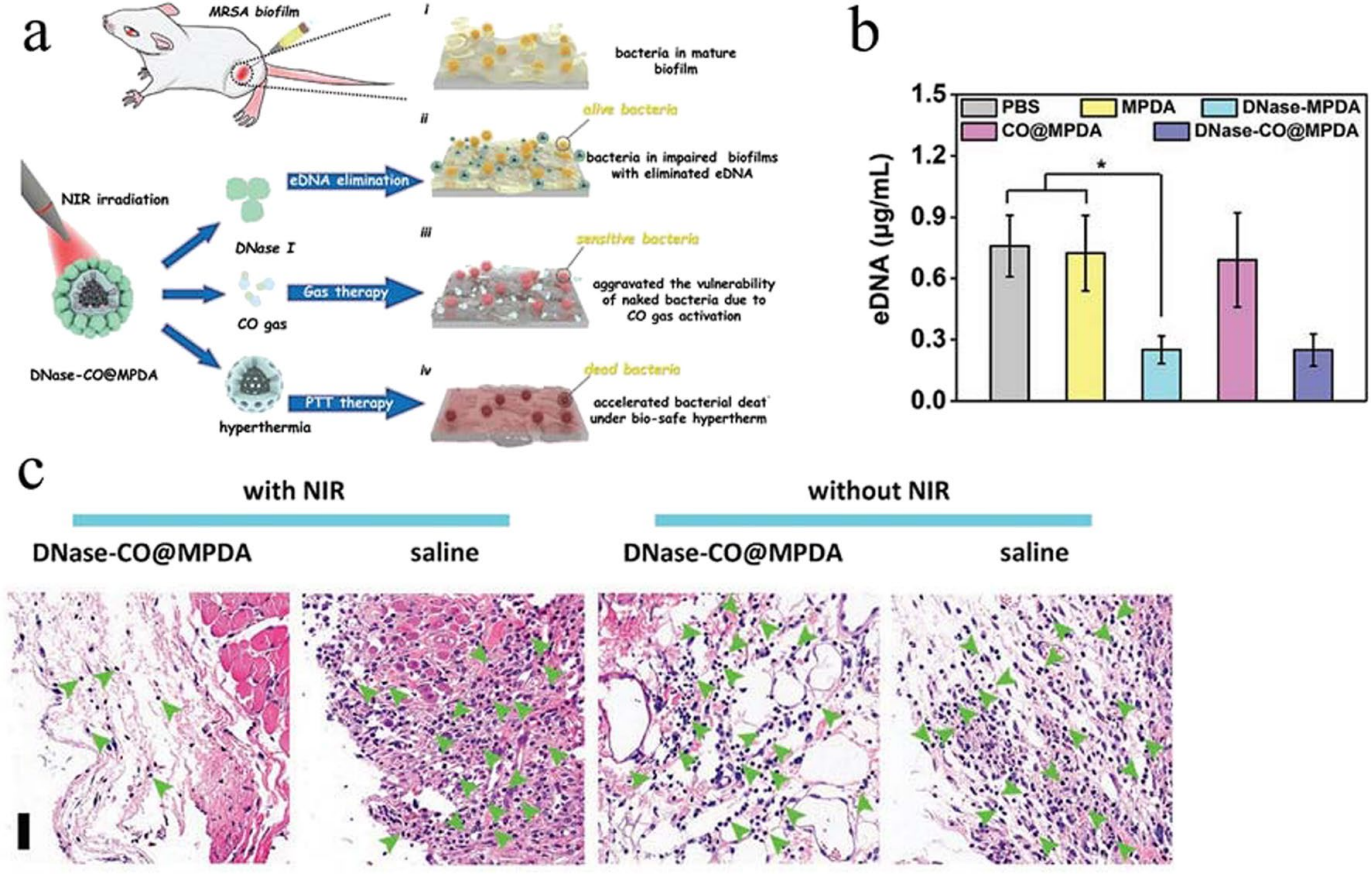
**a** Schematic diagram of DNase-CO@MPDA synergistic photothermal therapy (PTT) to enhance MRSA biofilm removal. **b** Residual amount of eDNA in MRSA biofilms after DNase-CO@MPDA treatment. **c** Histological micrographs of DNaseCO@MPDA-treated infected skin. Reprinted with permission from Ref. (Yuan et al. 2021) Copyright 2021, Wiley

Enzymatic dispersants break the three-dimensional structure of EPS by degrading extracellular polysaccharides, proteins, and eDNA within the biofilm. However, it has a poor bactericidal effect, often combined with the bactericidal ability of antimicrobial agents, which is a powerful countermeasure to eradicate biofilm and avoid reinfection. Currently, enzyme dispersants are generally modified on the surface of nanoparticles in a non-covalent manner to exert the desired effect. For example, serine proteases are electrostatically adsorbed on the surface of polyacrylic acid nano gels encapsulating ciprofloxacin (Cip) to enhance the impact of Cip on bacterial activity by hydrolyzing EPS (Weldrick et al. 2019). It is well known that novel nanoparticles such as Au, Ag, and Zn kill bacteria by converting heat energy by light or triggering the production of ROS, among which Ag nanoparticles have been used as broad-spectrum antimicrobial agents (Parham et al. 2016; Kim et al. 2018). DNase-functionalized gold nanoparticles (DNase-AuNCs) were designed (Xie et al. 2020). DNase combined with Ag phototherapy to achieve the desired effect of biofilm removal. Similar results have been achieved with invisible aligners, hinting at potential applications in medical devices. In addition, enzymatic dispersants can also modify the surface of nanoparticles by covalent means. It is well documented that carbon monoxide (CO) gas has a bactericidal effect by a mechanism related to targeting the bacterial respiratory chain to enhance ROS production and has good anti-inflammatory properties (Davidge et al. 2009; Motterlini and Otterbein 2010). DNase binds to the surface of polydopamine nanoparticles (MPDA) loaded with CO donor (FeCO) via Schiff base reaction or Michael addition reaction to form DNase-CO@MPDA nanoparticles (Fig. 4a) (Yuan et al. 2021). DNase-MPDA significantly disassembled the eDNA in the biofilm and achieved the disruption of the dense structure of the MRSA biofilm (Fig. 4 b). the NIR-triggered CO rapidly penetrates the biofilm’s interior and affects the bacteria’s viability. Under NIR-treated conditions, DNase-CO@MPDA reduced the inflammatory response and promoted wound healing by removing MRSA biofilm from abscess wounds, hindering the risk of systemic infection (Fig. 4c).

#### D-amino acid modified nanoparticles

D-AA may promote biofilm dispersion by affecting the structure of bacterial cell walls (De Pedro et al. 2003; Pidgeon and Pires 2017). The D-AA-modified nanoparticles report to have the property of dispersing biofilm, which removed the barrier for the adequate performance of antimicrobial agents (Johansson et al. 2011; Wei et al. 2015). D-cysteine (D-cys)-functionalized silver nanoparticles embedded in dopamine-coated stainless steel surfaces exert synergistic “dispersing and bactericidal” effects (Huang et al. 2020). D-cys not only inhibit biofilm formation but also disperse the physical barrier of biofilm so that Ag kills the dispersed bacteria and prevents further infection. Environmentally sensitive nanoparticles are widely designed to improve the selectivity of dispersants and drug release. Fan, Q. et al. attached D-Ty to CA-Tyr by cis-aconitic bonding and then bound electrostatically to the surface of polymeric micelles to form CM/Cy5.5@Tyr (Fan et al. 2021). When an acidic microenvironment reaches, the breakage of the acid-unstable cis-aconitine bond leads to the release of D-Ty with accompanying charge reversal that disperses and penetrates the dense biofilm to enhance the antibacterial effect of AZM. The study shows that the decomposition capacity of biofilms reached 82%, almost destroying the three-dimensional structure of biofilms. Treatment of lung infections has shown a reduction in the number of bacteria in the lungs and a decrease in fibrosis.

#### Rhamnolipid modified nanoparticles

**Fig. 5:**
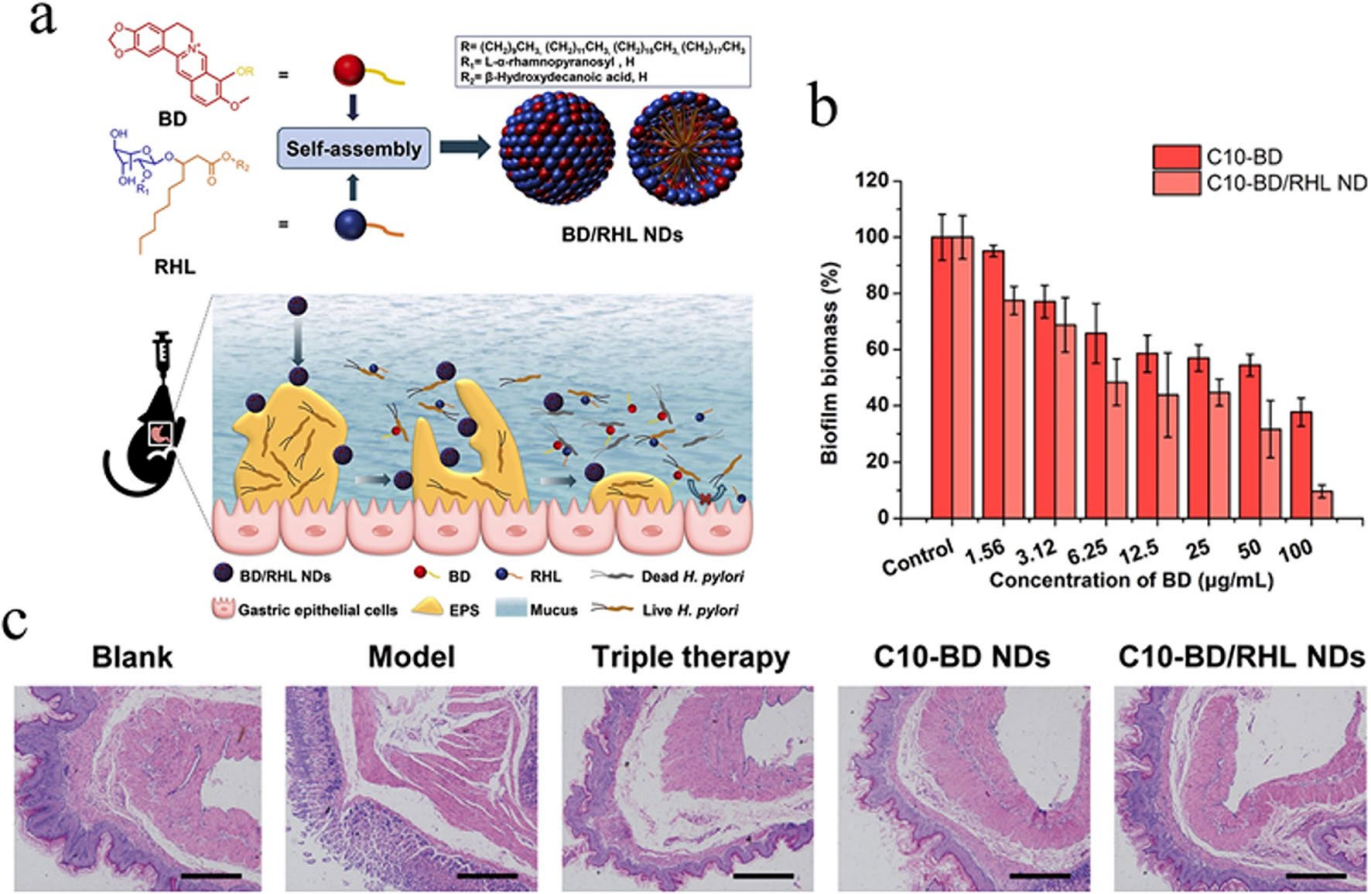
**a** Schematic diagram of the preparation of BD/RHL NDs and the mechanism of *H. pylori* biofilm remova. **b** Residual amount of *H. pylori* biofilm after BD/RHL NDs treatment. **c** Histological micrographs of *H. pylori* gastritis treated with BD/RHL NDs. Reprinted with permission from Ref. (Shen et al. 2020) Copyright 2020 Elsevier

RHL has the advantages of low toxicity, excellent biocompatibility, and removal of mature biofilms (Irie et al. 2005; Silva et al. 2017). The frequent use of RHL as a drug delivery vehicle has been reported (Niaz et al. 2019; Marangon et al. 2020). Li, P. et al. formed nanoparticles (PEG/CLR/RHL LPNs) loaded with clarithromycin (CLR) by amphiphilic self-loading of RHL and liposomes (Li et al. 2019). The mucus barrier biofilm model shows nanoparticles can effectively penetrate mucus, disperse *H. pylori* biofilm, and exert CLR antibacterial activity. Multifunctional self-assembled nanospheres (BD/RHL NDs) then prepare using lipophilic alkyl berberine derivatives (BDS) and RHL to overcome the dual barrier of the mucus layer and biofilm for the treatment of *H. pylori* gastritis (Fig. 5a) (Shen et al. 2020). In in vitro experiments, RHL-modified drug-loaded microspheres removed bacterial biofilms more effectively than drug-loaded microspheres alone, with 100 μg/mLC10-BD/RHL NDs removing 90.4% of bacterial biofilms (Fig. 5b). C10-BD/RHL NDs in treating H. pylori infection in the stomach reduced the number of *H. pylori* to nearly 0.6 log10 as measured by qPCR, with no abnormalities in the gastric mucosa. At the same time, the other groups had varying degrees of severe infiltration of inflammatory cells (Fig. 5c). RHL modified nanoparticles are powerful in eradicating *H. pylori* biofilms and reducing the damage caused by the inflammatory response associated with bacterial infection, thus cutting off a critical step in the recurrence of biofilm infections.

### Nanoparticles loaded with dispersants and antimicrobial agents

To avoid the destruction of dispersants by the immune system in vivo, loading dispersants into nanoparticles is often a standard means of effective delivery to the site of infection (Islan et al. 2015; Tan et al. 2018). Lipid nanoparticles loaded with levofloxacin (LV) and DNase for pulmonary delivery are a new alternative to improve current CF infections (Islan et al. 2016). DNase can reduce the viscoelasticity of lung mucus in CF patients, which contributes to the diffusion of LV and thus enhances the antimicrobial activity of LV. In addition, nanoparticles loaded with nano enzymes are also a standard form. For example, Yan, Z. et al. encapsulated CaO_2_ and heme-containing graphene (H-G) into alginate (Yan et al. 2018). It converted H_2_O_2_ to ROS by a local cascade reaction at the site of bacterial infection. It disrupted the main components of the biofilm (bacteria, polysaccharides, proteins, and nucleic acids), dispersing 81.5% of the biofilm and almost killing *S. aureus* in the biofilm. After 7 days of treatment, it killed more than 90% of the bacteria on the implanted catheter and promoted wound crusting and healing in the rats. Combining dispersants and antimicrobial agents through NDDS is an effective strategy to encourage physical barrier dispersion and hinder new infections caused by bacteria.

## Summary and challenges

The biofilm forms a complex physicochemical barrier that protects the encapsulated bacteria from antimicrobial drug treatment to some extent, further promoting the development of bacterial drug resistance. Dispersants are an effective means of breaking down physical barriers. Nanoparticles with stimulated response release dispersant to increase the dispersant accumulation at the infection site. It facilitates the ability to exert biofilm decomposition, which is a prerequisite for further antimicrobial action. However, its intelligence, stability, and sensitivity need further improvement. The bacterial biofilm consists of the inner bacteria and the outer layer of EPS. Bacterial biofilms treated with dispersants can expose the bacteria inside and, if left untreated, can cause new infections. So far, antimicrobial agent delivery by dispersant-based nanoparticles achieved the desired effect. It is an effective means of eradicating bacterial biofilms and alleviating recurrent infections caused by dispersed bacteria. However, the intrinsic cytotoxicity of nanomaterials remains a challenging issue, limiting further clinical applications of nanomaterials in biofilm infection-related diseases. Therefore, a more in-depth and careful examination of nanomaterials' long-term safety and biocompatibility is an important task.

## Data Availability

Data sharing is not suitable for this paper because no new data was created.
